# Association of Panton Valentine Leukocidin (*PVL*) genes with methicillin resistant *Staphylococcus aureus* (MRSA) in Western Nepal: a matter of concern for community infections (a hospital based prospective study)

**DOI:** 10.1186/s12879-016-1531-1

**Published:** 2016-05-15

**Authors:** Dharm R. Bhatta, Lina M. Cavaco, Gopal Nath, Kush Kumar, Abhishek Gaur, Shishir Gokhale, Dwij R. Bhatta

**Affiliations:** Central Department of Microbiology, Tribhuvan University, Kathmandu, Nepal; Research Group for Genomic Epidemiology, National Food Institute, Technical University of Denmark, Kgs Lyngby, Denmark; Department of Microbiology, Institute of Medical Sciences, Banaras Hindu University (BHU), Varanasi, India; Department of Microbiology, Manipal College of Medical Sciences, Pokhara, Nepal

**Keywords:** *Staphylococcus aureus*, MRSA, *PVL*, PCR

## Abstract

**Background:**

Methicillin resistant *Staphylococcus aureus* (MRSA) is a major human pathogen associated with nosocomial and community infections. Panton Valentine leukocidin (*PVL*) is considered one of the important virulence factors of *S. aureus* responsible for destruction of white blood cells, necrosis and apoptosis and as a marker of community acquired MRSA. This study was aimed to determine the prevalence of *PVL* genes among MRSA isolates and to check the reliability of *PVL* as marker of community acquired MRSA isolates from Western Nepal.

**Methods:**

A total of 400 strains of *S. aureus* were collected from clinical specimens and various units (Operation Theater, Intensive Care Units) of the hospital and 139 of these had been confirmed as MRSA by previous study. Multiplex PCR was used to detect *mecA* and *PVL* genes. Clinical data as well as antimicrobial susceptibility data was analyzed and compared among *PVL* positive and negative MRSA isolates.

**Results:**

Out of 139 MRSA isolates, 79 (56.8 %) were *PVL* positive. The majority of the community acquired MRSA (90.4 %) were *PVL* positive (Positive predictive value: 94.9 % and negative predictive value: 86.6 %), while *PVL* was detected only in 4 (7.1 %) hospital associated MRSA strains. None of the MRSA isolates from hospital environment was found positive for the *PVL* genes. The majority of the *PVL* positive strains (75.5 %) were isolated from pus samples. Antibiotic resistance among *PVL* negative MRSA isolates was found higher as compared to *PVL* positive MRSA.

**Conclusion:**

Our study showed high prevalence of *PVL* among community acquired MRSA isolates. Absence of *PVL* among MRSA isolates from hospital environment indicates its poor association with hospital acquired MRSA and therefore, *PVL* may be used a marker for community acquired MRSA. This is first study from Nepal, to test *PVL* among MRSA isolates from hospital environment.

**Electronic supplementary material:**

The online version of this article (doi:10.1186/s12879-016-1531-1) contains supplementary material, which is available to authorized users.

## Background

*Staphylococcus aureus* is one of the most common and important human pathogen associated with broad spectrum of diseases. It is a major cause of hospital acquired infection of surgical wounds and infections associated with indwelling medical devices. Increasing drug resistance among *S. aureus* and the spread of methicillin resistant *Staphylococcus aureus* (MRSA) are global threat. The resistance of MRSA to β-lactam antibiotics is associated with penicillin-binding protein 2a, encoded by the *mecA* gene. The pathogenicity of *S. aureus* is related to a number of virulence factors that allow the organism to adhere, avoid the immune system and cause harmful effects to the host. One of the important cytotoxins produced by some strains of *S. aureus* is the Panton Valentine leukocidin (*PVL*), encoded by two genes, *lukS- PV* and *lukF-PV* [1]. The Panton Valentine leukocidin was named after Sir Philip Noel Panton and Francis Valentine who associated it with soft tissue infections in 1932 [2, 3]. It is a member of the synergohymenotropic toxin family that induces pores in the membranes of cells.

Panton Valentine leukocidin producing MRSA usually cause mild skin or soft tissue infections, however, severe cases of necrotizing pneumonia and sepsis have also been reported [4]. Panton Valentine leukocidin is present in majority of community associated MRSA isolates and rarely present in hospital isolates, therefore it is recognized as marker of community acquired strains [5]. Epidemiological data suggest that high virulence of community acquired MRSA is associated with *PVL* genes but direct evidence of association of *PVL* to pathogenesis has been limited [6]. The prevalence of *PVL* genes among MRSA isolates has not been adequately reported from Nepal. This study was planned to investigate the prevalence of *PVL* genes among community and hospital- acquired MRSA isolates and to compare drug resistant pattern of *PVL* positive and *PVL* negative isolates. Isolates obtained from samples collected from the hospital environment including intensive care units were also included in this study in order to compare the association of *PVL* with MRSA isolates associated to the hospital environment.

## Methods

This prospective study was conducted at Microbiology laboratory of Manipal Teaching Hospital, Pokhara, Nepal, from September 2012 to August 2013. A total of 400 isolates of *S. aureus* had been collected in previous study [7] and 139 of these isolates had been confirmed as MRSA by susceptibility testing and PCR. These isolates were obtained from clinical specimens of various departments of the hospital (Surgery, Medicine, Intensive Care Units, Post-operative, Burn, Pediatric and Ear Nose Throat units). Isolates obtained from environmental samples collected from operation theaters and Intensive care units (ICU) were also included.

Isolation and identification of the isolates was performed by standard methods [8]. Antibiotic susceptibility testing was performed by Kirby-Bauer disc diffusion method [9] in previous study and data obtained was used for analysis. Minimal inhibitory concentration (MIC) of vancomycin was performed to rule out the possibility of vancomycin resistant *Staphylococcus aureus* (VRSA) and vancomycin intermediate *Staphylococcus aureus* (VISA) following CLSI guidelines [8]. *Staphylococcus aureus* showing resistance to at least one agent from three or more antimicrobial categories are labelled as multidrug resistant [10].

Hospital and community associated *S. aureus* isolates were categorized based on the following criteria: Isolates cultured from clinical specimens that were obtained after 72 h of admission of the patients or from patients with a history of hospitalization within 6 months were considered as hospital-acquired *S. aureus* strains; Isolates which were cultured within 72 h of hospitalization, from outpatient department (OPD) or patients with no history of hospitalization within 6 months were categorized as community- acquired strains. The clinical information on the patients’ clinical background which was used to set the criteria for classification of community and hospital acquired MRSA was obtained from the medical records.

### Detection of *mecA* and *PVL* genes by multiplex PCR

DNA was extracted from the MRSA isolates by chloroform: phenol extraction method as described by Sambrook et al. [11]. The primers used for *mecA* gene were **MecA1** (5′-GTA GAA ATG ACT GAA CGT CCG ATA A) and **MecA2** (5′-CCA ATT CCA CAT TGT TTC GGT CTA A) as described earlier by Geha et al. [12]. Primers used for detection of *PVL* genes were **Luk-PV-1** (ATC ATT AGG TAA AAT GTC TGG ACA TGA TCC A) and **Luk-PV-2** (GCA TCA AGT GTA TTG GAT AGC AAA AGC) which amplify a 433 base pair fragment specific for *lukS/F –PV* genes, encoding the *PVL* S/F bicomponent proteins as described by McClure et al. [13]. The DNA thermocycler was programmed for initial denaturation at 94 °C for 4 min; 30 cycles of amplification (denaturation at 94 °C for 45 s, annealing at 56 °C for 45 s, and extension at 72 °C for 30 s); and a final extension at 72 °C for 2 min. To visualize, 10 μl of the PCR amplicon was loaded with dye in 1.2 % agarose gel containing ethidium bromide followed by electrophoresis at 100 V for 1 h and visualized by using UV transillumination at 310 nm. Fragments of DNA 310 bp corresponded with *mecA* gene and 433 bp corresponded amplification of a fragment to the *PVL* genes.

#### Data analysis

Data was analyzed by using Pearson’s Chi-square test. A *p*-value of <0.05 was considered statistically significant.

## Results

**Fig. 1:**
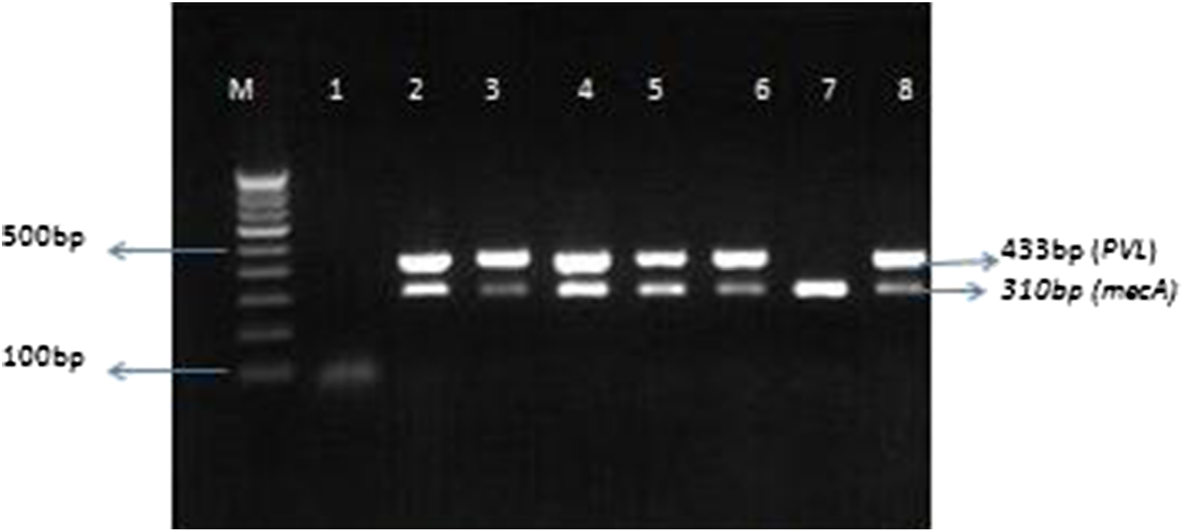
Multiplex PCR for *mecA* (310 bp) and *PVL* (433 bp) genes. *M*: Marker (100 bp), *1*: Negative control, *2*: Positive control, *3*–*8*: Test isolates

A total of 139 MRSA isolates from various clinical specimens were included in this study. Out of these, 35.2 % (49/139) were HA-MRSA, 59.7 % (83/139) were CA-MRSA and 5 % (7/139) were from hospital environment. The genes *mecA* (310 bp) and *PVL* (433 bp) were detected by multiplex PCR (Fig. 1). *MecA and PVL* genes were detected in 79/139 (56.8 %) of the isolates. The majority of the *PVL* positive isolates were obtained from pus samples accounting for 74/98 (75.5 %). The remaining sample types showed lower percentage of *PVL* genes whereas among the MRSA from hospital environment samples, none of the isolates were found positive for *PVL* (Table 1).

**Table 1:** Distribution of *PVL* genes among MRSA isolates in different specimens

Specimen type	Total number of MRSA	*PVL* positive (%)
Pus	98	74 (75.5 %)
Blood	14	2 (14.3 %)
Urine	12	2 (16.6 %)
Sputum	6	1 (16.6 %)
Hospital environment	7	0
Body fluids	2	0

The results of antimicrobial susceptibility testing of 139 isolates are shown in Table 2. Our analysis could not find any statistically significant differences in the susceptibility pattern of *PVL* positive and *PVL* negative isolates except towards erythromycin. Seventy three percent (102/139) MRSA were multidrug resistant; 50 were *PVL* positive while 52 were *PVL* negative. Among the 79 *PVL* positive isolates, 63.3 % (50/79) were MDR, while this percentage was found significantly higher 86.6 % (52/60) among *PVL* negative isolates (*p* value: <0.005).

**Table 2:** Antibiotic resistance pattern of *PVL* positive and *PVL* negative MRSA isolates

Antibiotic	*PVL* positive MRSA (*n* = 79) Frequency (%)	*PVL* negative MRSA (*n* = 60) Frequency (%)	*P* value
Penicillin	79 (100 %)	60 (100 %)	–
Erythromycin	52 (65.8 %)	50 (83.3 %)	0.021^a^
Ciprofloxacin	61 (77.2 %)	51 (85 %)	0.251
Cotrimoxazole	48 (60.7 %)	45 (75 %)	0.077
Gentamicin	34 (43 %)	32 (53.3 %)	0.229
Clindamycin	08 (10.1 %)	07 (11.6 %)	0.772
Amikacin	06 (7.6 %)	08 (13.3 %)	0.266
Tetracycline	04 (5 %)	06 (10 %)	0.265
Vancomycin	00	00	–

^a^Significant association

Among 37 MRSA isolates which were non MDR, 29 (78.4 %) were *PVL* positive and 8 (21.6 %) were *PVL* negative. The difference between *PVL* positive non MDR and *PVL* positive MDR MRSA was statistically significant (*p* value <0.001).

Out of the 139 MRSA isolates, 56 (40.3 %) were found to be hospital associated MRSA and the remaining 83 (59.7 %) isolates were community associated MRSA by above mentioned clinically based criteria. Among the 83 CA-MRSA, 75 (90.4 %) were *PVL* positive while only 7.1 % (4/56) HA-MRSA were *PVL* positive (*p* value <0.001). All seven MRSA isolates obtained from environmental samples were negative for *PVL*.

## Discussion

Global emergence of MRSA is serious public health problem and challenge to clinicians. A number of factors contribute to the pathogenicity and drug resistance of *S. aureus*. The first *PVL* positive MRSA was observed in the late 1990 and these strains have become globally distributed in the recent years [14]. The role of *PVL* in enhancing virulence of *S. aureus* and their pathogenicity is being debated. Panton Valentine leukocidin increases the pathogenicity of *S. aureus* by necrosis, accelerating apoptosis and destruction of polymorphonuclear and mononuclear cells thereby contributing to morbidity and mortality [15]. However, some studies have shown no association of *PVL* with the virulence of the organism by demonstrating better clinical outcome of skin and soft tissue infections [16, 17]. Therefore, the role of *PVL* in clinical outcome is still debated. The reason for the results in clinical outcomes in these studies could be influenced by the effectiveness of antibiotic treatment applied.

Panton Valentine leukocidin is commonly used as a marker for community acquired MRSA, responsible for soft-tissue and deep dermal infections [18, 19]. However, the global scenario of *PVL* among MRSA isolates varies.

Reports from various countries show the increasing prevalence of *PVL* among MRSA isolates [20, 21]. Subarna Roy et al. from India, have reported overall 62.85 % of *PVL* prevalence among MRSA and MSSA (MRSA: 85.1 % and MSSA: 48.8 %) which indicates a higher prevalence among MRSA than our findings [22]. Similar study by D’Souza et al. from Mumbai, India, reported prevalence of 64 % *PVL* positive isolates among MRSA [23]. A lower prevalence of *PVL* has been reported from other parts of world (5 % in France, 4.9 % in UK, 8.1 % in Saudi Arabia and 14.3 % in Bangladesh) [15, 24–26], reflecting that the prevalence of *PVL* varies greatly between geographical locations and populations.

This study analyzes the role of *PVL* in infections at different sites. Skin and soft tissue infections are predominantly (75.5 %) caused by *PVL* producing organisms as the leucocidal activity of *PVL* provides survival advantage to the bacteria. The association of *PVL* with isolates from other specimens was less. Presence of *PVL* in deep seated infections like blood stream infections was found less common in our study indicating poor association of *PVL* with invasiveness of MRSA.

The results of antimicrobial susceptibility testing revealed higher resistance among *PVL* negative MRSA isolates as compared to *PVL* positive MRSA isolates, however the differences were not statistically significant except in case of erythromycin (Table 2). Similar finding was observed in case of clindamycin and tetracycline in another study from Nepal [27].

Similarly, the percentage of MDR MRSA among *PVL* negative (86.6 %) isolates was found significantly higher than in *PVL* positive (63.3 %) isolates (*p* value <0.005). These findings suggest that *PVL* is probably not associated with MDR phenotypes in this study. Similarly, a significantly higher prevalence of *PVL* was observed among non MDR MRSA.

Association of *PVL* among male patients was found slightly higher (54 %) than female (46 %) patients. Higher prevalence of *PVL* among children (<14 years of age) was observed as compared to adults and old age group patients, although difference was statistically insignificant. Similar findings were observed in another study from India [28]. However, some studies have reported strong association of *PVL* among young children [29]. The highest number (65/79) of *PVL*-MRSA were isolated from the patients of surgery department, followed by the burn units (5/79), the orthopedic unit (5/79), and other departments (4/79). Similar distribution of *PVL* positive MRSA isolates in various units of hospital was reported from India [28].

*PVL* was considered as important marker for differentiation of HA-MRSA and CA-MRSA. In our study, 75 out of 83 CA-MRSA isolates were found *PVL* positive with positive predictive value 94.9 % and negative predictive value 86.6 %. However, some studies have shown association of *PVL* genes among HA- MRSA isolates also [27, 28]. Most of the studies including our study categorized HA-MRSA and CA-MRSA based upon the history of the patient or by getting information from medical record. However, information obtained from patient or from medical record may not be reliable all the time. To overcome this, we for the first time from Nepal, tested seven isolates of MRSA obtained from the environment of various units of the hospital including wards and intensive care units. As these isolates are not known to be related to the specimen from patients and isolated from hospital environment, we considered them as presumptive hospital strains. These seven isolates were found negative for *PVL* genes which could indicate that *PVL* is not normally found in the isolates of hospital environment. However, these isolates are not necessarily representative of hospital environment in general. Absence of *PVL* in *S. aureus* from inanimate objects of hospital environment may indicate limited role of antileucocytic activity outside the host. In our study, we found association of *PVL* gene in four MRSA isolates which are hospital acquired as per the clinical criteria described above. As the majority of *PVL* positive strains represent community isolates, this shows that the criteria set for this study might have limitations and/or these isolates could have been originated from out patients and transmitted to health care workers and patients. To our knowledge, it is likely that these four *PVL* positive isolates might have been recently transmitted from a community source to hospital settings. In contrast, another study from Nepal reported higher prevalence of *PVL* among nosocomial MRSA isolates [27].

Limitation of the study: *SCCmec* typing was not performed in the current study but is planned to be pursued in further studies.

## Conclusion

The prevalence of the *PVL* among the MRSA isolates in this study was found relatively high especially among pus samples which indicate a possible key role of *PVL* in pathogenesis of pyogenic infections especially skin and soft tissue infections in community setting. The *PVL* positive MRSA isolates showed higher sensitivity against antibiotics as compared to *PVL* negative isolates indicating that *PVL* is not associated with drug resistance mechanisms. The presence of *PVL* among multi drug resistant bacteria like MRSA may be involved in virulence and increase the challenges for clinicians. As expected, the majority of *PVL* positive MRSA were community-associated isolates, whereas only four MRSA from hospital related cases were found positive for *PVL*. No *PVL* was detected in MRSA isolated from the hospital environment. In our view, the presence of *PVL* can be used as a reliable marker for CA-MRSA in these resource limited settings in Nepal.

### Ethics approval and consent to participate

Ethical approval to conduct the study was obtained from the Institutional Ethical Committee (IRC), Manipal College of Medical Sciences (MCOMS), Pokhara, Nepal. Consent of patients was not required as samples were taken as a routine part of care.

### Availability of data and materials

Data supporting the findings can be found in the tables. Data supporting the absence of *PVL* genes among hospital environmental isolates can be found in the Additional file 1: Figure S1 (Isolate number 20–26 are MRSA isolated from hospital environment showed absence of *PVL* genes).

## Additional file

Additional file 1: Figure S1. Seven MRSA isolates (Sample number 20–26) from hospital environment showing absence of *PVL* genes. (DOCX 118 kb)

## Abbreviations

°C: degrees celsius
CA-MRSA: community acquired methicillin resistant *Staphylococcus aureus*
CLSI: clinical and laboratory standards institute
HA-MRSA: hospital acquired methicillin resistant *Staphylococcus aureus*
MDR: multidrug resistant
MIC: minimal inhibitory concentration
MRSA: methicillin resistant *Staphylococcus aureus*
PCR: polymerase chain reaction
*PVL*: panton valentine leukocidin
VISA: Vancomycin intermediate *Staphylococcus aureus*
VRSA: Vancomycin resistant *Staphylococcus aureus*
