# Supplementary material for: Association of Panton Valentine Leukocidin (PVL) genes with methicillin resistant Staphylococcus aureus (MRSA) in Western Nepal: a matter of concern for community infections (a hospital based prospective study)
Source: BMC Infect Dis. 2016 May 15;16:199. doi: 10.1186/s12879-016-1531-1 (PMC4867903; doi:10.1186/s12879-016-1531-1)
Supplement: Supplementary file 1 [file 12879_2016_1531_MOESM1_ESM.docx]

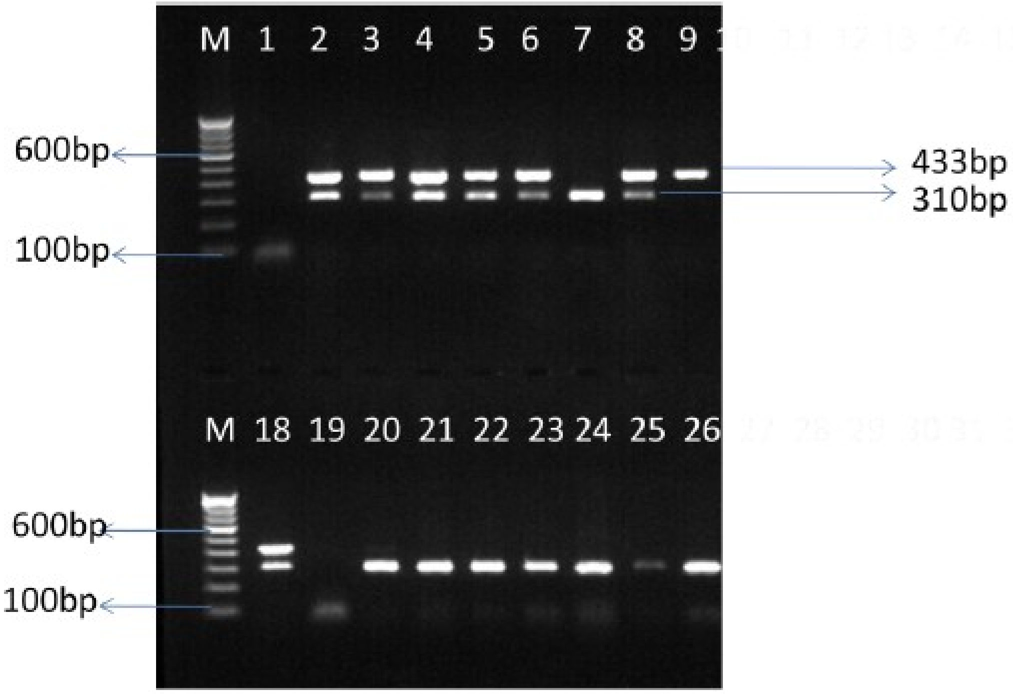
M 1 2 3 4 5 6 7 8 9
600bp
100bp
433bp
310bp
M 18 19 20 21 22 23 24 25 26
600bp
100bp
